# Minimally Invasive Posterolateral Approach for Surgical Resection of Dumbbell Tumors of the Lumbar Spine

**DOI:** 10.3389/fsurg.2022.792922

**Published:** 2022-02-09

**Authors:** Talgat T. Kerimbayev, Zhandos M. Tuigynov, Viktor G. Aleinikov, Yermek A. Urunbayev, Yergen N. Kenzhegulov, Dinara M. Baiskhanova, Nurzhan B. Abishev, Meirzhan S. Oshayev, Makar P. Solodovnikov, Serik K. Akshulakov

**Affiliations:** JSC “Center for Neurosurgery”, Nur-Sultan, Kazakhstan

**Keywords:** lumbar spine, dumbbell tumor, minimally invasive, posterolateral approach, resection

## Abstract

Minimally invasive spine surgery (MISS) has many advantages over traditional open surgical procedures that can be conducted for the therapy of different diseases of the spine. MISS provide many prospective advantages such as, for example, small incisions, less damage to soft tissues, early activation of patients, and a shorter postoperative hospital stay. The aim of the study was to evaluate institutional experience with Dumbbell tumors and metastatic lesions of the lumbar spine and compare it with traditional open surgical resection of this type of tumors. Fourteen patients underwent the surgery with minimally invasive posterolateral approach in experimental group, and 10 patients of the control group were operated using the traditional open surgery procedure at the Department of spinal neurosurgery and pathology of peripheral nervous system of JSC “National Center for Neurosurgery.” The intraoperative neuro monitoring system (ISIS IOM System Compact, Inomed, Germany) was used in both groups. Sensory and motor evoked potentials were intraoperatively recorded. The present study was approved by the local Ethics Committee of the National Center for Neurosurgery. Patients signed informed consent before the surgical procedure. The experimental group included 14 patients, that underwent the surgery during the period from January 2020 till March 2021. And the control group included 10 patients that was operated from January 2018 to December 2019. The results of the treatment in both groups were assessed according to the generally accepted visual analog scale (VAS) and the Oswestry scales before, on the third day, and 3 months after the surgery. In experimental group, average reduction of the pain syndrome of 3.36 points (from 3 to 0 points) was observed in patients postoperatively according to the VAS 3 days, and of 4.0 points (from 2 to 0 points) 3 months after surgical procedures. Improvement by 23.86% (36–16%) was also observed using the Oswestry Disease Index (ODI) 3 days after the surgery, and then reduced to 21.00% (16–34%) in average in 3 months. All patients were revived 3 h after transfer to the specialist department. The average stay in the hospital was 6.5 (9–4) days in both groups. In control group, average reduction of the pain syndrome of 2.60 points (from 4 to 1 points) was observed postoperatively according to the VAS 3 days after the operation, and of 3.9 points (from 2 to 0 points) 3 months after the surgery. The ODI of patients was also improved by an average of 35.40% (50–20%) 3 days after the surgical procedure, and reduced to 24.20% (16–32%) in average 3 months after the surgery.

## Introduction

Minimally invasive spine surgery (MISS) is an alternative to traditional open surgical procedures performed for the treatment of various diseases of the spine, such as osteochondrosis, herniated disc, scoliosis, spinal stenosis, and tumors. MISS offer many potential benefits, such as small incisions, less damage to soft tissues (ligaments, muscles), early activation of patients, and a shorter postoperative hospital stay (1, 2). Nowadays, there is a possibility (if necessary) to stabilize the functional spinal unit (FSU) using the percutaneous technique of introducing transpedicular screws.

Among spinal neoplasms, the incidence of dumbbell tumors is 13–14% of those occupying the perforaminal location, while in 41% of cases, tumors are observed in the cervical spine. The traditional surgical approach for the removal of such type of tumors involves an extended skin incision with dissection of soft tissues and extensive skeletonization of the muscle layer; resection of the arches and facet joints of the vertebrae. This, in turn, potentially cause instability in the involved FSU.

**Figure 1:**
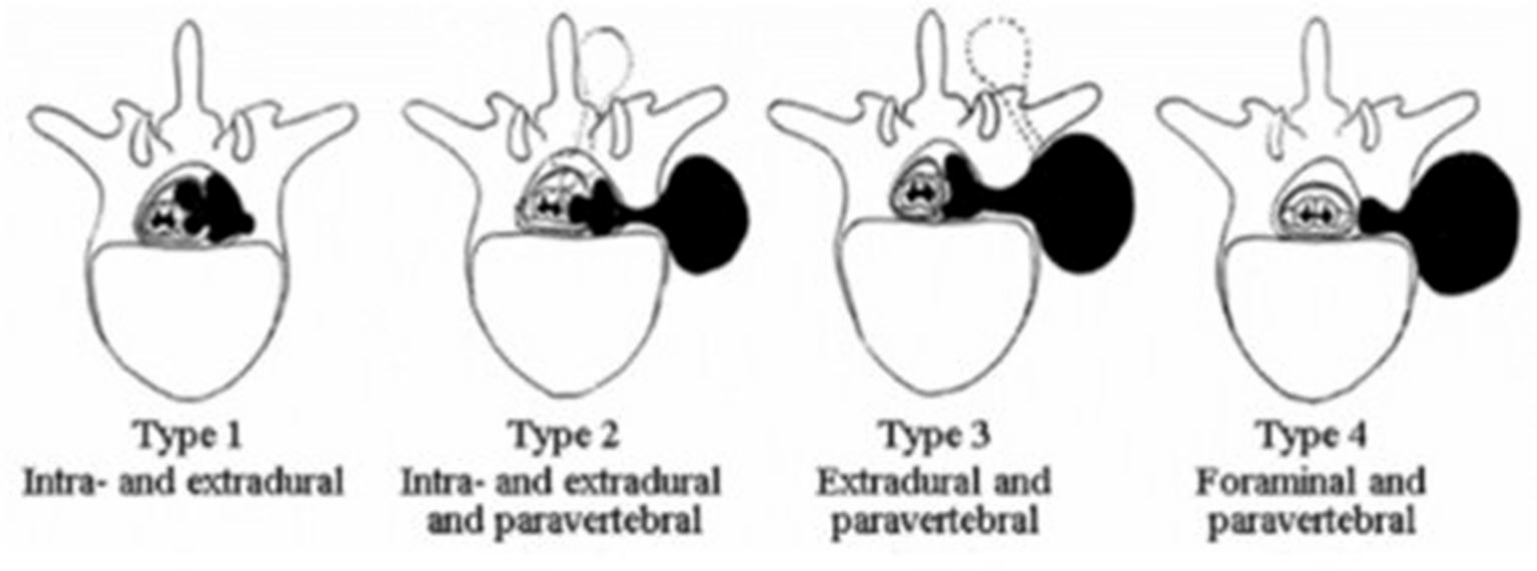
Eden's classification of spinal dumbbell tumors, from left to right: type I–rumors with intra- and extradural components; type II–with intra-, extradural, and paravertebral components, type III–with extradural and paravertebral components, type IV–with foraminal and paravertebral components.

In 1941, Eden proposed a classification in which tumors are systematized depending on their topographic and anatomical interrelation with the nerve and bone structures of the spine. However, it does not provide an answer regarding the size of neoplasms. According to the literature, the most common type of tumors is type III tumors with extradural and paravertebral components according to the Eden's classification (Figure 1) (3). Metastatic lesions of the spinal cord and the spine can also occur in the perforaminal location—a common complication of cancer disease. Damage of the spine and roots of the spinal cord can significantly reduce the quality of life of patients, potentially causing persistent pain (4). Due to the early detection and an increase in the life expectancy of patients with malignant tumors, the number of patients at risk of developing metastases is increasing every year (5, 6). According to the statistics, the spine is the third most frequent region of cancer cell metastasis after the lungs and liver. And it is expected, that nearly 70% of cancer patients will have metastases to the spine. In the case of symptomatic lesions, the majority of metastases (60–70%) are found in the thoracic region, while the remaining 20% are in the lumbar region and 10% in the cervical spine. More than 50% of patients with spinal metastases have more than one lesion level (7, 8).

The goal of surgical intervention for spinal metastases is palliative care in the form of pain reduction and improvement of the quality of life in patients with pain syndrome. In some cases, if necessary, stabilization of the spinal motion segments is performed. Moreover, one of the goals of the surgery is the collection of biopsy material for subsequent histological and immunohistochemical studies. In order to reduce postoperative complications and speed up postoperative recovery of patients, minimally invasive approaches may be the best technique of surgical intervention. Satisfactory decompression and minor surgical aggression are critical for patients with concomitant conditions and untreated accompanying decompensated disease that prevents comprehensive surgery (9). Recent advances in microsurgical techniques have led to the development of minimally invasive approaches for the treatment of primary and metastatic spinal lesions. This, in turn, results in reduced postoperative pain, shorter overall hospital stay, reduced blood loss during surgery, improved neurological status, and earlier initiation of adjuvant therapy (10, 11). These benefits are particularly crucial for maintaining and improving the quality of life of cancer patients with a short life expectancy (12, 13). The MISS methodology aims to perform: a minimally invasive posterolateral tubular access to remove the tumor and decompress the spinal cord, reducing intraoperative blood loss and postoperative pain.

Yet, there is some criticism of this technique in the form of difficulties in achieving sufficient decompression of the spinal cord. This is the result of the mistaken belief that the greater the surgical exposure, the better the results are achieved. On the contrary, in fact, MISS techniques provide facilitated access to the spinal canal and complete decompression of the spinal cord and its roots (14, 15).

## Materials and Methods

The present study is a consistent retrospective single-center case control series study. Cases of dumbbell tumors and metastatic lesions of the lumbar spine were included in this study. In the experimental group, 14 patients underwent surgery with minimally invasive posterolateral approach (Table 1), and 10 patients in control group were operated using the traditional open surgery technique (Table 2) at the Department of spinal neurosurgery and pathology of peripheral nervous system of JSC “National Center for Neurosurgery.” Intraoperative neuro-monitoring system (ISIS IOM System Compact, Inomed, Germany) was used in both groups. Sensory and motor evoked potentials were intraoperatively recorded. The present study was approved by the local Ethics Committee of the National Center for Neurosurgery. Patients signed informed consent before surgical procedure. Experimental group included 14 patients, that underwent the surgery during the period from January 2020 till March 2021. And the control group included 10 patients that was operated from January 2018 to December 2019. Two groups was consecutive case series. Data on the experimental group are highlighted in Table 1, and data on the control group are presented in Table 2. Diagnosis and preoperative assessment were performed using magnetic resonance imaging (MRI) with contrast enhancement of the lumbar spine. Patients with tumor sizes >8.0 cm in the largest diameter according to MRI data were excluded from this study in two groups. Evaluation of the stability of FSU was performed using functional X-ray images. The results of the treatment in both groups were assessed according to the generally accepted the VAS and the ODI scales before the surgery, on the third day, and 3 months after the surgerical procedures. Before the surgical operation, all patients underwent hormonal preoperative preparation with intravenous dexamethasone according to the scheme of 16 mg per day for 2 days. All patients were administered non-steroidal anti-inflammatory drug after the surgical procedure 8–16 mg intramuscularly daily depending on the VAS of a patient. We used standard surgical technique that is described below in the chapter “Description of the surgical procedure” to reduce inter- or intra-operation variations, ensure the quality, and maintain consistency between cases. The follow-up period after the surgery of all patients was 3–12 months (6.5 months on the average) with contrast MRI of the lumbar spine 3 days and 3 months after surgical procedures.

**Table 1:** Results of performed surgery on 14 patients (Experimental group).

**Gender/age (y)**	**The level of lumbar spine lesions**	**Type of a tumor according to Eden's classification**	**VAS before the surgery**	**Oswestry before the surgery**	**Histology**	**VAS after the surgery**	**VAS after 3 month surgery**	**Ostwery after the surgery**	**Ostwery after 3 month surgery**	**The number of days spent in stasis unit**	**Required instrumentation (fusion)**	**Blood loss**	**Operation time**
1. Fem/33	L2–L3 left side	3	3	38	Schwannoma	0	0	18	18	5	No	30	115
2.Fem/45	L4–L5 right side	3	5	50	Schwannoma	2	2	24	20	7	No	35	80
3.Fem/74	L3–L4 right side	4	7	74	Mts	3	2	34	34	9	No	110	150
4.Male/51	L1–L2 left side	4	4	42	Schwannoma	0	0	24	18	6	No	45	90
5.Male/60	L2–L3 left side	4	3	40	Schwannoma	1	1	18	16	5	No	50	85
6.Fem/65	L1–L2 left side	4	6	68	Mts	2	1	36	28	7	No	30	125
7.Fem/45	L3–L4 right side	3	4	48	Schwannoma	1	1	22	18	5	No	35	100
8.Male/42	L4–L5 left side	4	4	50	Schwannoma	1	0	22	18	6	No	50	120
9.Fem/32	L2–L3 right side	4	2	44	Schwannoma	0	0	16	16	4	No	35	105
10.Male/69	L1–L2 right side	4	6	64	Mts	2	1	32	28	8	No	65	100
11.Fem/50	L2–L3 right side	3	4	46	Schwannoma	1	1	20	20	7	No	50	140
12.Male/41	L2–L3 right side	3	3	40	Schwannoma	0	0	18	18	7	No	30	90
13.Male/62	L3–L4 left side	4	6	66	Schwannoma	1	0	26	22	8	No	35	80
14.Fem/54	L4–L5 left side	4	4	62	Schwannoma	0	0	24	20	7	No	30	95

**Table 2:** Results of performed surgery on 10 patients (Control group).

**Gender/age (y)**	**The level of lumbar spine lesions**	**Type of a tumor according to Eden's classification**	**VAS before the surgery**	**Oswestry before the surgery**	**Histology**	**VAS after the surgery**	**VAS after 3 month surgery**	**Oswestry after the surgery**	**Oswestry after 3 month surgery**	**The number of days spent in stasis unit**	**Requared instrumentaion (fusion)**	**Blood loss**	**Operation time**
**1**.Male/25	L3–L4 left side	3	4	40	Schwannoma	1	0	20	16	9	No	150	90
**2**.Male/62	L3–L4 left side	4	6	66	Schwannoma	2	0	44	32	10	Yes	170	120
**3**.Fem/35	L4–L5 right side	3	4	64	Schwannoma	2	1	50	30	10	No	120	90
**4**.Fem /50	L2–L3 right side	4	5	46	Schwannoma	2	0	36	22	9	No	80	100
**5**.Male/40	L1–L2 left side	3	4	40	Schwannoma	1	0	20	16	7	No	70	120
**6**.Male/60	L2–L3 right side	4	6	50	Mts	2	1	36	30	6	No	80	80
**7**.Fem/43	L4–L5 right side	4	5	56	Schwannoma	2	1	24	20	7	No	100	70
**8**.Fem /48	L3–L4 left side	3	4	54	Schwannoma	3	1	40	22	10	No	90	90
**9**.Male/40	L1–L2 left side	4	4	40	Schwannoma	2	1	36	22	8	No	70	100
**10**.Male/60	L1–L2 right side	3	4	60	Schwannoma	4	2	48	32	12	No	110	90

### Description of the Surgical Procedure

#### Intervention Details

**Figure 2:**
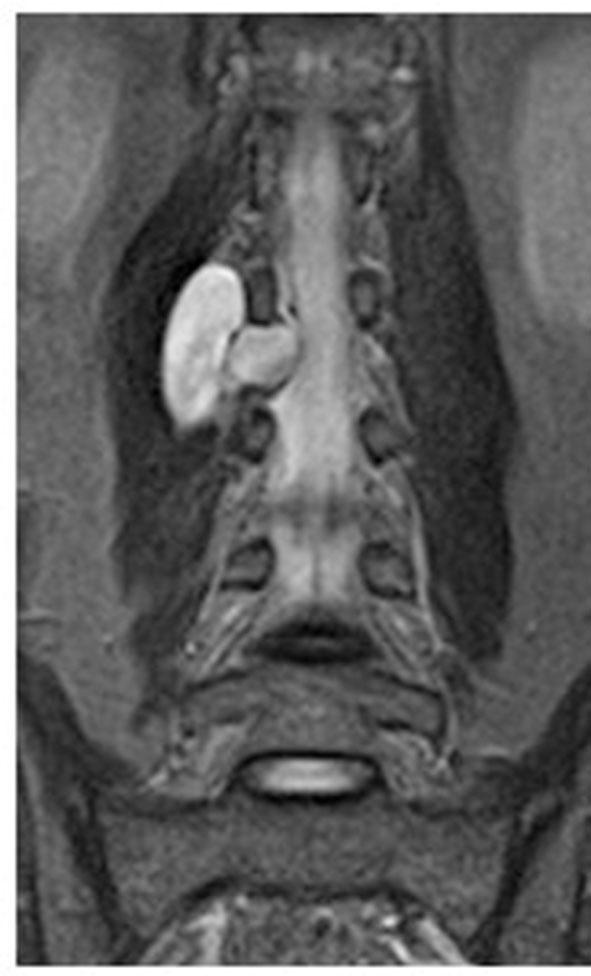
Preoperative contrast-enhanced MRI of the lumbar spine.

**Figure 3:**
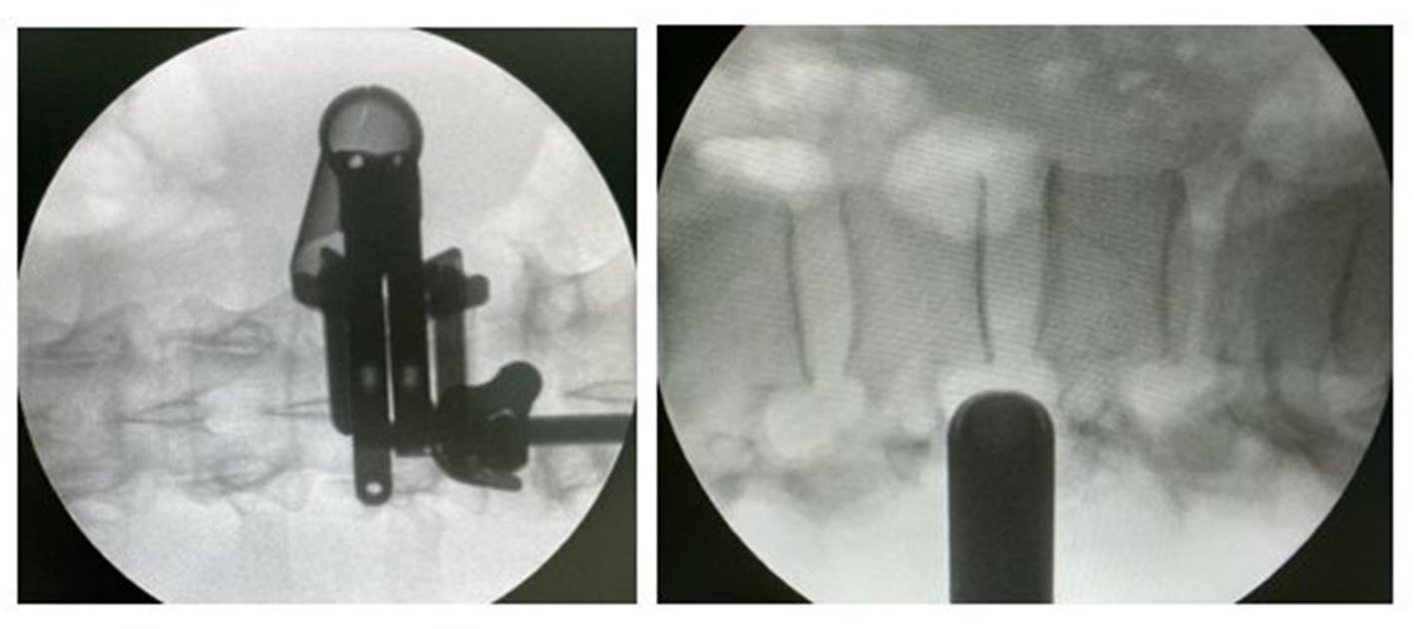
Direct and lateral X-ray control during the surgery.

**Figure 4:**
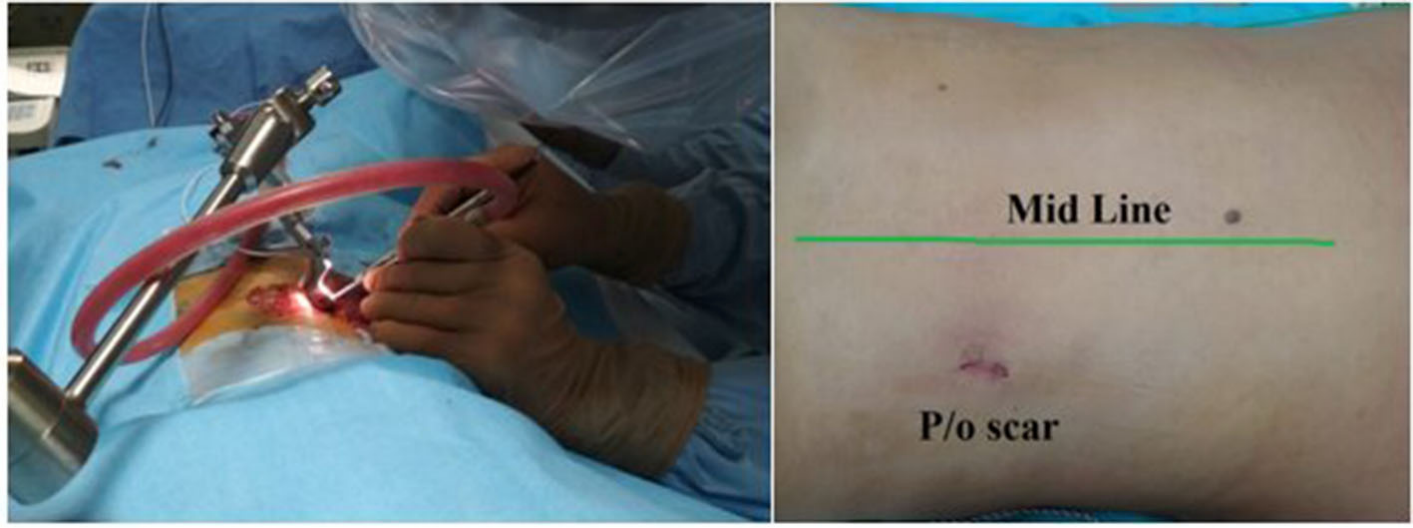
Left side–Intraoperative picture; right side–postoperative scar (2 cm).

**Figure 5:**
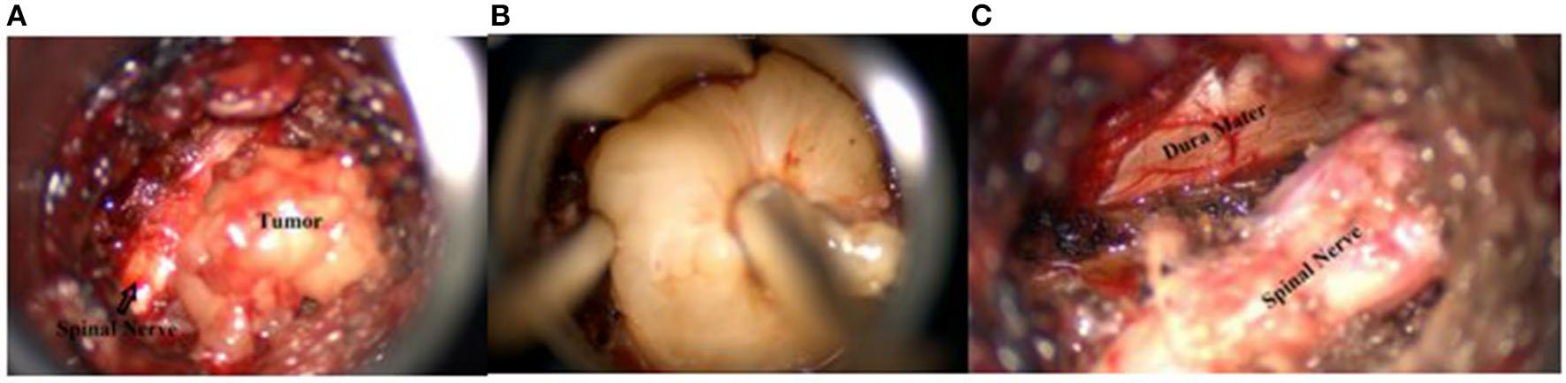
**(A)** The rumor and compressed emerging nerve root (picture obtained from the operating microscope); **(B)** lntraoperative photograph: removal of the tumor; **(C)** After the removal of the tumor, the dura mater and root are visualized (photograph from the operating microscope).

**Figure 6:**
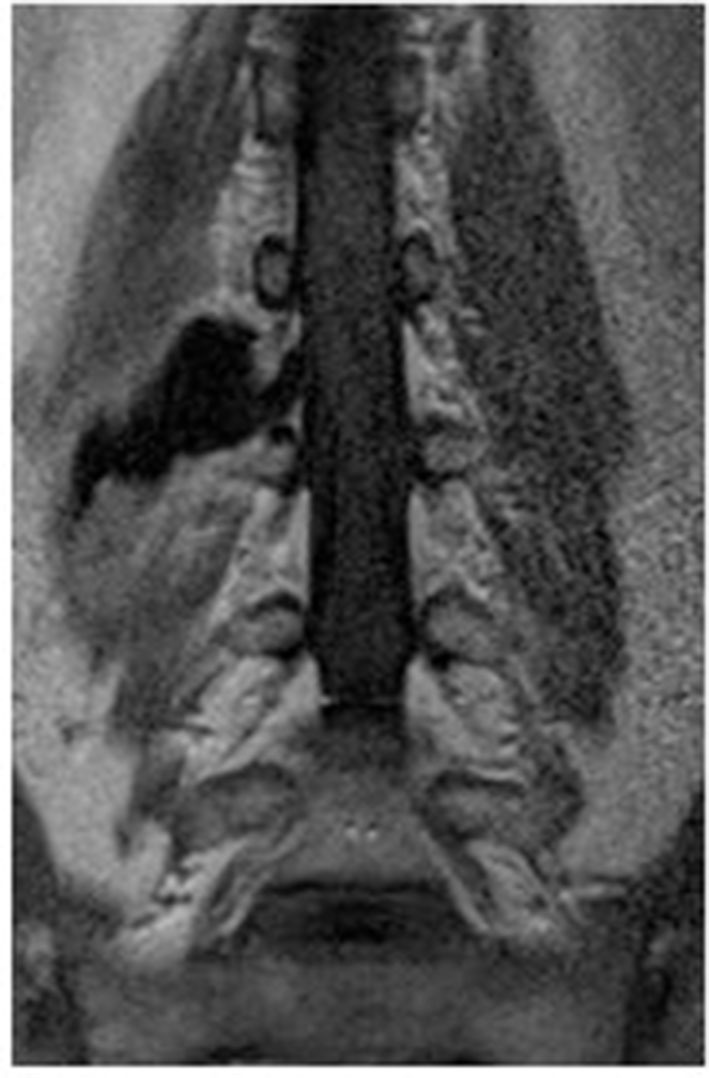
MRI of the lumbar spine with contrast after the surgery.

All surgical manipulations on removing tumors and metastatic lesions were performed *via* minimally invasive posterolateral access using the Mast Quadrant Tubular Retractor System (Medtronic Sofamor Danek, Memphis, TN, USA). All patients underwent the surgery with IONM with recording of sensory and motor signals from the lower limbs. Patients were administered general anesthesia before the surgical procedure, and then laid on a multifunctional operating table, in a supine position with pelvic rollers under the shoulders and with the arms brought forward. Intraoperative fluoroscopy was used to determine the level of surgical intervention. All 24 surgical procedures in two groups (the experimental group and the control group) were performed by three senior neurosurgeons, with the experience more than 15 years in the spine surgery. A minimally invasive posterolateral tubular approach was performed through a linear skin incision about 2.0 cm long (1.5–2.5 cm), extending from the supraspinous line (paraspinally) to the width of the paraspinal muscles (5.0–8.0 cm) at a sufficient angle to expose the ipsolateral extraforaminal space of the affected region. The subcutaneous fatty tissue and muscle layer were “dilated” using a tubular system, under fluoroscopic control (straight and lateral spondylograms). The handle of the tubular dilator was rigidly attached to the operating table using a holder. Further imaging was performed under an operating microscope (HS 5–1,000, Haag-Streit Surgical, Germany) at up to 24-x magnification. The space between the transverse processes of the adjacent vertebral segments was found and partially resected with Kerrison-type 2.0 bone excisors, when it was necessary. The extraforaminal components of the tumor were isolated using microinstrumentation, and when it was necessary, removal was performed using an ultrasound aspirator. The exiting nerve root was completely exposed, and the dura mater was identified. During the surgical operation the nerve root was protected using a “holder.” The foraminal opening was completely cleared of the tumor and decompression was performed. Tumor tissue was sent for the histological analysis. Thorough hemostasis was performed at all stages of the surgery. Fascia and aponeurosis were sutured in layers with interrupted sutures. The tubular retractor was removed. Then, a cosmetic, atraumatic, continuous suture was applied to the skin. A drainage tube was installed into the wound cavity when it was necessary. Figures 2–6 illustrate case examples of the surgical removal of a tumor with every stage of the surgery.

### Statistical Analysis of Variables Between Two Groups Is Presented in Table 3

^*^p-values had been calculated using chi-square test or Fisher's exact test for categorical variables, while Mann-Whitney *U*-test had been used for numeric variables. Comparison of variables between two groups.

**Table 3:** Comparison of variables between two groups.

**Variables**	**Control (*n* = 10)**	**MISS (*n* = 14)**	***p*-value**
Gender, Male (%)	6 (60.0)	6 (42.9)	0.68
Age (years)			
Median (IQR)	45.5 (40–57.5)	50.5 (42.8–61.5)	0.32
Mean (SD)	46.30 (12.07)	51.64 (12.99)	
The level of lumbar spine lesions (%)			0.84
L1–L2	3 (30.0)	3 (21.4)	
L2–L3	2 (20.0)	5 (35.7)	
L3–L4	3 (30.0)	3 (21.4)	
L4–L5	2 (20.0)	3 (21.4)	
VAS before the surgery			
Median (IQR)	4 (4–5)	4 (3.3–5.8)	0.64
Mean (SD)	4.60 (0.84)	4.36 (1.45)	
Oswestry before the surgery			
Median (IQR)	52 (41.5–59)	49 (42.5–63.5)	0.88
Mean (SD)	51.60 (9.97)	52.29 (12.05)	
Histology, Schwannoma (%)	9 (90.0)	11 (78.6)	0.85
VAS after the surgery			
Median (IQR)	2 (2–2)	1 (0–1.8)	0.01
Mean (SD)	2.10 (0.88)	1.00 (0.96)	
VAS after 3 month surgery			
Median (IQR)	1 (0–1)	0.5 (0–1)	0.85
Mean (SD)	0.70 (0.67)	0.64 (0.74)	
Ostwery after the surgery			
Median (IQR)	36 (27–43)	23 (18.5–25.5)	<0.01
Mean (SD)	35.40 (10.92)	23.86 (6.25)	
Ostwery after 3 month surgery			
Median (IQR)	22 (20.5–30)	19 (18–21.5)	0.19
Mean (SD)	24.20 (6.29)	21.00 (5.31)	
The number of days spent in stasis unit			
Median (IQR)	9 (7.3–10)	7 (5.3–7)	<0.01
Mean (SD)	8.80 (1.81)	6.50 (1.40)	
Required instrumentation (fusion), Yes (%)	1 (10.0)	0 (0.0)	0.86
Blood loss			
Median (IQR)	95 (80–117.5)	35 (31.3–50)	<0.001
Mean (SD)	104.00 (34.06)	45.00 (21.48)	
Operation time			
Median (IQR)	90 (90–100)	100 (90–118.8)	0.22
Mean (SD)	95.00 (15.81)	105.36 (21.88)	

## Results

All 14 patients in the experimental group underwent single-stage minimally invasive posterolateral approach with the follow-up period from 3 to 12 months (6.5 months on the average). Other characteristics of patients related to gender, age, diagnosis, and comorbidities are highlighted in Table 1. According to Eden's classification, 5 (35.7%) patients had type III of spinal dumbbell tumor, and 9 (64.3%) patients had type IV of the tumor. The most patients (5 patients, 35.7%) of the experimental group underwent surgical resection of L2–L3 vertebrae. Total tumor removal was achieved in 12 patients (85.7%), and subtotal tumor removal was performed in 2 patients (14.3%) with cases of comprehensive metastatic lesions. In the experimental group, radiological assessment of the stability of the involved FSU was performed postoperatively (X-ray with functional tests of the lumbar spine), and stabilization was not required in this group. The surgical operation lasted 80–150 min (mean−105.36 min) with blood loss of 30–110 ml (mean−45.0 ml). Histological analysis revealed schwannomas in 11 (78.6%) patients, while undifferentiated carcinoma metastases was observed in 3 (21.4%) patients. No recurrence was observed in schwannomas during the follow-up period. The main preoperative indicators such as the VAS and the ODI scores in both groups were 4.60 and 4.36, 51.60 and 52.29% on average, respectively. There were no significant intraoperative or postoperative complications in our series of all 24 patients in both groups. The neurological status of all patients in the postoperative period remained without worsening of sensory and motor responses in the extremities compared to the preoperative state (as indicated by IONM). It could be due to the adequate visualization of the nerve structures and the use of IONM. In experimental group, the average reduction of the pain syndrome of 3.36 points (from 3 to 0 points) was observed according to the VAS 3 days in the postoperative period, and of 4.0 points (from 2 to 0 points) 3 month after the surgical operation. The same picture was also observed using the ODI on the third day after the surgery [23.86% on the average (36–16%)]. Moreover, this score reduced to 21.00% (16–34%) on the average in 3 months. All patients were revived 3 h after transfer to the specialist department. The average hospital stay was 6.5 (9–4) days on the average. Regarding the control group, reduction of the pain syndrome was also observed in patients postoperatively: the VAS was 2.60 points (from 4 to 1 points) on the average 3 days after the surgical procedure, and 3.9 points (from 2 to 0 points) 3 months after the surgery. Enhancement was also observed in ODI [35.40% (50–20%) on average] in 3 days, and reduced to 24.20% (16–32%) on the average 3 months after the operation. The results of the performed surgery in control group are shown in Table 2.

## Discussion

This study produced promising findings concerning minimally invasive posterolateral approach for tumors of the spinal cord and its roots, comparing to the traditional laminectomy with resection of the arches and facet joints of the vertebrae. As an example, in one of the cases of the study in a control group, the surgical procedure on stabilization of the spinal motion segment on the level of L3–L4 vertebrae was performed in a patient. The patient had the pain syndrome in the lumbar region as a result of instability that was confirmed on control radiographs with functional tests. This procedure was carried out to stabilize the functional spinal unit using percutaneous technique of introducing transpedicular screws. As a result of the stabilization, the pain syndrome totally regressed during 3 months postoperatively.

On the contrary, patients of the experimental group did not require additional stabilizing surgery, that contribute to the preservation of the arch and the facet joint in the stability of the spinal motion segment postoperatively in patients with this kind of spinal cord tumors.

There is a conviction that surgical resection of dumbbell tumors of the spinal cord and its roots is always challenging. Yet, based on our own experience, we can still claim that minimally invasive posterolateral approach is a worthy alternative to the traditional surgical procedures in resection of such types of tumors in the spinal cord and its roots. For example, nowadays, minimally invasive decompression and stabilization methods are widely used by spinal surgeons and linked to more optimistic clinical results due to reduced paravertebral tissue injury, minimum postoperative pain syndrome, and shorter patient's recovery time after the operation (1, 2). The minimally invasive posterolateral approach for tumors of the spinal cord and its roots has many advantages in contrast to the traditional laminectomy. Firstly, there is no traction on nerve structures, which in turn contribute to prevention of post-operative neurological complications. Secondly, it facilitates to preservation of the ligamentary apparatus further maintaining movement ability in the functional segment. Moreover, the most important benefit of the MISS is the complete removal of the spinal cord tumor. Lastly, minimally invasive posterolateral approach promotes early postoperative rehabilitation of patients. As a result, patients can have the possibility to take an upright posture the same day after the surgical procedure without additional external immobilization devices. This is confirmed in the present study by comparison of both groups of patients (Table 3). In the MISS group, more rapid improvement was observed in the ODI when assessed on the 3rd day after the surgery (23.86 points on the average) comparing to the data in the control group that was 36.40 points on the average. Nevertheless, the leveling of the difference of this indicator by the third month was also noted on the average 24.20 points in the MISS group, and 21.00 points in the control group (Table 4).

**Table 4:**
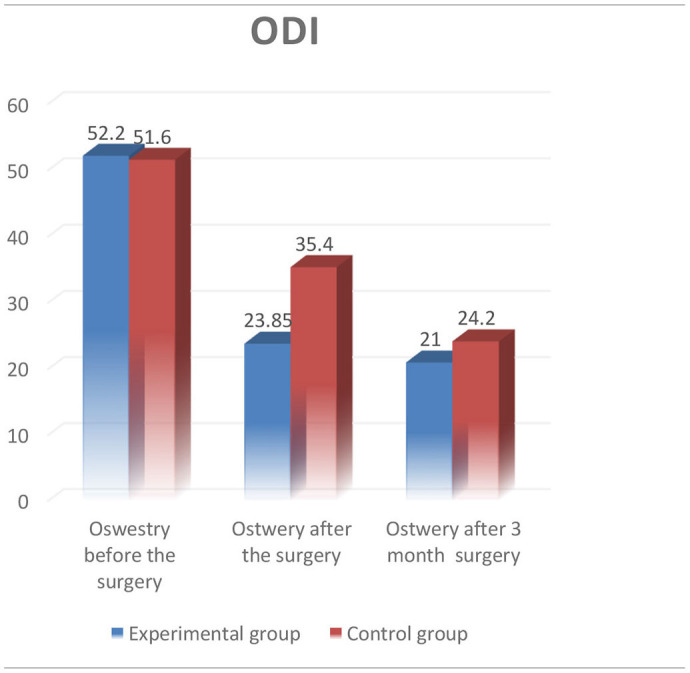
Comparison ODI.

However, the difficulties of using MISS to decompress sufficiently the spinal cord, in spinal cord tumors still remain controversial. On the contrary, MISS techniques provide easy access to the spinal cord and its roots, as well as their complete decompression, if necessary. In comparison to the traditional laminectomy allows to decrease the volume of blood loss by a patient, and to reduce significantly the pain syndrome after the surgery according to the VAS.

In our experimental group, the decrease in the VAS was 3.36 units after the surgical procedure, and 4.0 units 3 months after the surgery comparing to the control group, where the decrease in the VAS after the operation was 2.6 and 3.9 units on the average 3 months after the surgical procedure. Based on the obtained data, we can expect a faster reduction in the pain syndrome in patients when using the minimally invasive posterolateral approach, by 1.0 point on the average within 3 days after the surgery (Table 5).

**Table 5:**
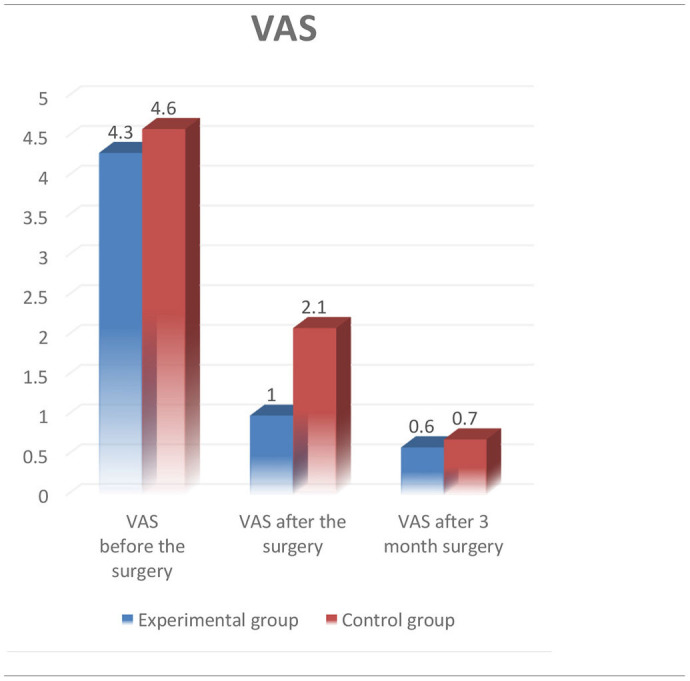
Comparison VAS.

In addition, the somatic status of patients was considerably improved. Nevertheless, further prospective research including larger amount of patients with longer follow-up period is of a strong need in order to compare various results representing the effectiveness and lack of side effects of microsurgical techniques compared to traditional open surgical procedures.

## Data Availability Statement

The original contributions presented in the study are included in the article/supplementary material, further inquiries can be directed to the corresponding author.

## Ethics Statement

The studies involving human participants were reviewed and approved by Ethics Committee of the National Center for Neurosurgery, November 2019. The patients/participants provided their written informed consent to participate in this study. Written informed consent was obtained from the individual(s) for the publication of any potentially identifiable images or data included in this article.

## Author Contributions

TK: conceptualization. ZT: data collection. TK, VA, YU, and NA: surgical procedures. MO, NA, and YK: postoperative observation of patients. TK, ZT, and VA: writing draft. DB, MS, and SA: review and editing of manuscript. All authors contributed to the article and approved the submitted version.

## Funding

All surgical procedures in this research were supported by the financial means of the Health Ministry of the Republic of Kazakhstan.

## Conflict of Interest

The authors declare that the research was conducted in the absence of any commercial or financial relationships that could be construed as a potential conflict of interest. The handling Editor declared a past co-authorship with one of the authors TK and SA.

## Publisher's Note

All claims expressed in this article are solely those of the authors and do not necessarily represent those of their affiliated organizations, or those of the publisher, the editors and the reviewers. Any product that may be evaluated in this article, or claim that may be made by its manufacturer, is not guaranteed or endorsed by the publisher.
